# Brain-constrained neural modeling explains fast mapping of words to meaning

**DOI:** 10.1093/cercor/bhad007

**Published:** 2023-02-20

**Authors:** Marika Constant, Friedemann Pulvermüller, Rosario Tomasello

**Affiliations:** Brain Language Laboratory, Department of Philosophy and Humanities, WE4, Freie Universität Berlin, Habelschwerdter Allee 45, 14195 Berlin, Germany; Faculty of Life Sciences, Department of Psychology, Humboldt-Universität zu Berlin, Unter den Linden 6, 10099 Berlin, Germany; Bernstein Center for Computational Neuroscience Berlin, Philippstraße 13 Haus 6, 10115 Berlin, Germany; Berlin School of Mind and Brain, Humboldt-Universität zu Berlin, Luisenstraße 56, 10117 Berlin, Germany; Brain Language Laboratory, Department of Philosophy and Humanities, WE4, Freie Universität Berlin, Habelschwerdter Allee 45, 14195 Berlin, Germany; Berlin School of Mind and Brain, Humboldt-Universität zu Berlin, Luisenstraße 56, 10117 Berlin, Germany; Einstein Center for Neurosciences Berlin, Charitéplatz 1, 10117 Berlin, Germany; Cluster of Excellence ‘Matters of Activity. Image Space Material’, Humboldt-Universität zu Berlin, Unter den Linden 6, 10099 Berlin, Germany; Brain Language Laboratory, Department of Philosophy and Humanities, WE4, Freie Universität Berlin, Habelschwerdter Allee 45, 14195 Berlin, Germany; Cluster of Excellence ‘Matters of Activity. Image Space Material’, Humboldt-Universität zu Berlin, Unter den Linden 6, 10099 Berlin, Germany

**Keywords:** language acquisition, fast mapping, semantic grounding, Hebbian learning, distributed neural assemblies, biologically neural networks

## Abstract

Although teaching animals a few meaningful signs is usually time-consuming, children acquire words easily after only a few exposures, a phenomenon termed “fast-mapping.” Meanwhile, most neural network learning algorithms fail to achieve reliable information storage quickly, raising the question of whether a mechanistic explanation of fast-mapping is possible. Here, we applied brain-constrained neural models mimicking fronto-temporal-occipital regions to simulate key features of semantic associative learning. We compared networks (i) with prior encounters with phonological and conceptual knowledge, as claimed by fast-mapping theory, and (ii) without such prior knowledge. Fast-mapping simulations showed word-specific representations to emerge quickly after 1–10 learning events, whereas direct word learning showed word-meaning mappings only after 40–100 events. Furthermore, hub regions appeared to be essential for fast-mapping, and attention facilitated it, but was not strictly necessary. These findings provide a better understanding of the critical mechanisms underlying the human brain’s unique ability to acquire new words rapidly.

## Introduction

Humans show a remarkable capacity to acquire a large vocabulary of meaningful symbols rapidly in early ontogeny. The ability to *instantaneously* map and store a novel word together with its related referent, known as “fast-mapping,” was first reported by Carey and Bartlett (1978). Although there is some evidence that animals are capable of fast-mapping to a limited extent (Kaminski et al. 2004), the widespread and automatic nature of this learning in young children suggests that it is a crucial mechanism for the fast vocabulary growth in early language development (von Koss Torkildsen et al. 2008). Examining fast-mapping at the neurophysiological level, studies have shown activity changes due to rapid word learning in young children (Friedrich and Friederici 2011), as well as surprisingly rapid neuronal changes in frontal–temporal–parietal regions after short word-meaning exposures in adults (Hofstetter et al. 2017). Similarly, Vukovic and colleagues recently reported microstructural changes in the lexical-semantic network during short sessions when learning novel object and action words (Vukovic et al. 2021). However, several aspects of a complete neuro-mechanistic account of this fast learning remain unclear.

In order to examine the brain mechanisms underlying language learning at the cellular and cortical level, a computational approach can be fruitful. Importantly, however, fast-mapping seemingly contrasts with learning algorithms used in most network simulations. Although these algorithms are inspired by biological learning, they typically take thousands of learning events to reliably store information (Lake et al. 2017; Devlin et al. 2019; Shorten and Khoshgoftaar 2019), which raises the question of how a mechanism for fast-mapping is realized in biological systems. Although we note that there are also neural networks optimized for learning speed (Regier 2005; Mayor and Plunkett 2010), this apparent contrast between fast vs gradual learning has led some cognitive scientists to suggest that, for explaining fast-mapping and other features of human language learning, substantial a priori knowledge is necessary (Mayor and Plunkett 2010; Atir-Sharon et al. 2015). For fast-mapping, this includes pre-existing mental representations of both entities in the external world and phonological structures. In fact, by the time infants start to understand language, they have already had extensive experience with objects and actions, as well as phonological forms via babbling or imitation (MacNamara 1972; Vihman et al. 1985; Werker and Tees 1999; Werker and Hensch 2015). The resultant phonological and conceptual representations may be key to establishing rapid associations between word forms and their referent representations, which is supported by neurophysiological research (Sharon et al. 2011; Smith et al. 2014; Atir-Sharon et al. 2015). Previous modeling efforts have aimed at capturing this principle with a two-stage semantic learning process involving an initial encoding of concepts followed by a second learning stage relying on this previously formed framework (Schyns 1991; Plunkett et al. 1992; Regier 2005; Mayor and Plunkett 2010). However, these models have either used neurophysiologically implausible learning rules (such as backpropagation) and/or have strayed from realistic neurophysiology and anatomy in other ways. Biological realism of neural networks is critical for exploring cognitive processing, as structural specificities of the human cortex may be crucial for supporting uniquely human capacities such as language processing (Pezzulo et al. 2013; Breakspear 2017; Pulvermüller et al. 2021; Schomers et al. 2017).

Following such a strategy, neurobiological models have been developed mimicking anatomical and physiological properties of frontal-temporal-occipital regions (Garagnani and Pulvermüller 2016; Tomasello et al. 2017, 2018, 2019; Henningsen-Schomers and Pulvermüller 2022). Although these models have captured semantic learning, it remains to be examined in detail when symbol-meaning associations first form and how they develop across learning. This investigation is needed in order to explore whether a biologically realistic learning rule such as Hebbian learning allows for an explanation of fast-mapping. Here, we aimed to clarify the cortical mechanisms of rapid word-meaning mapping in a biologically constrained spiking network model with 12 cortical areas essential for phonological processing, modality-specific visual and motor processing, and multimodal information integration. We focused on the grounded word learning mechanisms involved in fast-mapping in the context of object perception and action execution, in contrast to the disjunctive syllogism captured by the classical fast-mapping paradigm, in which children infer the referent of a new label by rejecting an alternative referent with a known label (Carey and Bartlett 1978). Specifically, we simulated key features of two different semantic association learning scenarios: with initial exposure to phonological and conceptual information separately, as claimed by fast mapping theory (two-stage learning), and without it, simulating direct word-meaning mapping (one-stage learning). We asked, within a biologically plausible cortical model with Hebbian correlation learning and connectivity structure based on neuroanatomical evidence, (i) whether it is possible to capture fast-mapping between phonological and referent representations, (ii) if the presence of pre-existing conceptual representations in the brain prior to semantic learning promotes fast-mapping, and (iii) which role attentional mechanisms play for fast-mapping.

## Materials and methods

### General model architecture

**Fig. 1:**
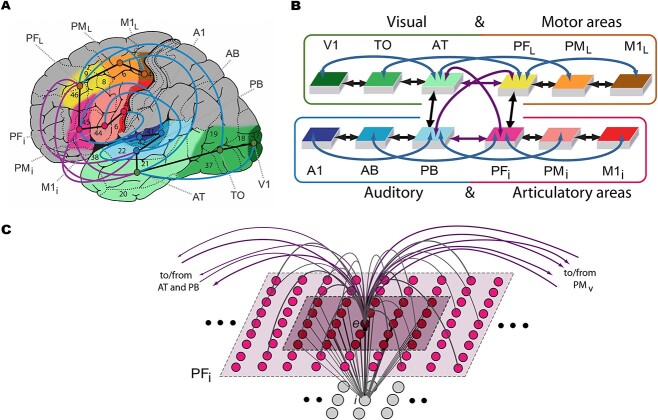
Model structure and connectivity. **A**) The structure and connectivity of the 12 network areas are shown, with the perisylvian articulatory-phonological system in red/pink colors, including primary motor cortex (M1_i_), premotor cortex (PM_i_), and inferior prefrontal cortex (PF_i_), and the acoustic phonological system in blue, including the primary auditory cortex (A1), auditory belt (AB), and parabelt (PB). Extrasylvian regions include the dorsolateral hand-motor system in yellow/brown, consisting of the lateral prefrontal (PF_L_), premotor (PM_L_), and primary motor (M1_L_) cortex, as well as the ventral visual stream in green, including the anterior temporal (AT), temporo-occipital (TO), and primary visual (V1) areas. Numbers refer to Brodmann areas and arrows between areas represent long distance cortico-cortical connections. **B**) Schematic of the areas and their connectivity structure. **C**) Micro-connectivity structure of one modeled excitatory “cell,” labeled *e*. Gray lines arching upward represent within-area excitatory links that are limited to the local neighborhood (light shaded area). Purple lines arching upward capture between-area links. The underlying gray cells represent an inhibitory cell *i*, which inhibits neighbors proportional to the total input it receives from the neighborhood shaded in darker purple. Figure adapted from Tomasello et al. (2018).

We used a brain-constrained spiking neural network of twelve cortical areas of the frontal, temporal, and occipital lobes relevant for language and semantic processing. Six of these areas are located in the perisylvian language cortex and are critical for the processing of spoken word forms (Fadiga et al. 2002; Pulvermüller and Fadiga 2010; MacGregor et al. 2012). These regions can be divided into two modality-preferential systems: articulatory, including inferior face-motor and -premotor along with inferior prefrontal regions; and auditory, including superior-temporal primary, belt, and parabelt areas (regions highlighted in blue and red, Fig. 1). The remaining six areas are extrasylvian regions relevant for referential meaning-related information and can be divided into the ventral (visual) processing stream, known to be involved in object perception and recognition (Ungerleider 1994), and the dorsolateral (motor) system, known to be involved in action execution (regions highlighted in green and brown/yellow, Deiber et al. 1991; Lu et al. 1994; Dum and Strick 2002, 2005).

Briefly, we modeled the following anatomical and physiological principles of the cerebral cortex, which have been argued to be critical to simulate higher cognitive functions (see Pulvermüller et al. 2014; Pulvermüller et al. 2021):

(i) neurophysiological dynamics of spiking pyramidal cells including temporal summation of inputs, threshold-based spiking, and adaptation (Connors et al. 1982; Matthews 2001);
(ii) synaptic plasticity by way of Hebbian-type learning, including both long-term potentiation (LTP) and depression (LTD, Artola and Singer 1993);
(iii) local lateral inhibition and area-specific regulation mechanisms (Braitenberg 1978; Yuille and Geiger 2003);
(iv) within-area connectivity based on a sparse, randomly initiated local connectivity with a neighborhood bias toward close-by links (Kaas 1997; Braitenberg and Schüz 1998);
(v) between-area connectivity based on neuroanatomical principles and motivated by neuroanatomical evidence further explained below (see also Table 1); and
(vi) presence of ongoing uniform uncorrelated white noise in all neurons during all phases of learning (Rolls and Deco 2010), and additional static noise added to the stimulus patterns to mimic realistic variability of input conditions during learning.

**Table 1:** Connectivity structure. References on which connectivity structure of the network model is based, divided by connection type and involved regions. Table taken from Tomasello et al. 2018.

**Modeled Areas**	**References**
**Connections between next-neighbor areas** (black arrows)	
*Perisylvian system*	
A1, AB, PB	Pandya 1995; Kaas and Hackett 2000; Rauschecker and Tian 2000
PF_i_, PM_i_, M1_i_	Pandya and Yeterian 1985; Young et al. 1994
*Extrasylvian system*	
V1, TO, AT	Bressler et al. 1993; Distler et al. 1993
PF_L_, PM_L_, M1_L_	Pandya and Yeterian 1985; Arikuni et al. 1988; Lu et al. 1994; Rizzolatti and Luppino 2001; Dum and Strick 2002, 2005
*Between system*	
AT, PB	Gierhan 2013
PF_i_, PF_L_	Yeterian et al. 2012
**Connections between second-next-neighbor areas** (blue arrows)	
*Perisylvian system* Rilling et al. 2008, 2012; Thiebaut de Schotten et al. 2012	
A1, PB	Pandya and Yeterian 1985; Young et al. 1994
PB, PMi	Rilling et al. 2008; Saur et al. 2008
AB, PFi	Romanski, Bates, et al. 1999; Kaas and Hackett 2000; Petrides and Pandya 2009; Rauschecker and Scott 2009
PFi, M1i	Deacon 1992; Young et al. 1994; Guye et al. 2003
*Extrasylvian system* Thiebaut de Schotten et al. 2012	
V1, AT	Catani et al. 2003; Wakana et al. 2004
AT, PM_L_	Bauer and Fuster 1978; Fuster et al. 1985; Pandya and Barnes 1987; Seltzer and Pandya 1989; Chafee and Goldman-Rakic 2000
TO, PF_L_	Bauer and Jones 1976; Fuster and Jervey 1981; Fuster et al. 1985; Seltzer and Pandya 1989; Makris and Pandya 2009
PF_L_, M1_L_	Deacon 1992; Young et al. 1994; Guye et al. 2003
**Long distance cortico-cortical connections** (purple arrows)	
*Perisylvian system*	
PF_i_, PB	Meyer et al. 1999; Romanski, Tian, et al. 1999; Paus et al. 2001; Catani et al. 2005; Parker et al. 2005; Rilling et al. 2008; Makris and Pandya 2009
*Extrasylvian system*	
AT, PF_L_	Bauer and Jones 1976; Ungerleider et al. 1989; Eacott and Gaffan 1992; Webster et al. 1994; Fuster 1995; Parker and Gaffan 1998; Chafee and Goldman-Rakic 2000
*Between system*	
PB, PF_L_	Pandya and Barnes 1987; Romanski, Bates, et al. 1999; Romanski, Tian, et al. 1999
AT, PF_i_	Pandya and Barnes 1987; Ungerleider et al. 1989; Webster et al. 1994; Romanski 2007; Petrides and Pandya 2009; Rilling 2014

The single-neuron properties, synaptic plasticity rule, and single-area model structure are specified in more detail below and in previous publications (Garagnani et al. 2017; Tomasello et al. 2018, 2019).

In order to simulate a fast-mapping learning mechanism, two learning phases were implemented. First, the model was trained with separate instances of word forms and referents, to mimic previous experiences a child would have had with objects, action execution, and production of meaningless phonological word forms prior to semantic learning (Jusczyk and Hohne 1997; Quinn et al. 1997; Quinn and Schyns 2003; Tsao et al. 2004; Kuhl et al. 2005; Swingley 2007). Subsequently, the model was presented with the learned auditory word form and referent information simultaneously to simulate associative semantic learning in which a word is heard, while the referent being spoken about is present (Tomasello and Kruger 1992; Vouloumanos and Werker 2009). This is in line with the proposed theory of fast-mapping (Mayor and Plunkett 2010; Sharon et al. 2011; Smith et al. 2014; Atir-Sharon et al. 2015), in which rapid word learning is based on the link between pre-existing phonological and referent knowledge representations. In addition, this learning process was compared to a one-step learning mechanism, in which the model was not exposed to any prior phonological or referent-related experience (simulating direct word-meaning association) in order to assess the importance of this pre-existing knowledge for learning speed. The methods section under “*Simulated learning*” explains in more detail how precisely the learning was undertaken.

### Structural model features

#### Neuron model

The artificial units used to approximate the function of pyramidal neurons in the cortex were integrate-and-fire neurons (Matthews 2001) whose synaptic connections were modified according to a Hebbian learning mechanism (Hebb 1949). The neural dynamics included several properties of pyramidal cells, such as the temporal summation of excitatory and inhibitory inputs, “all-or-nothing” threshold-based spiking, and neuronal adaptation based on a cell’s recent firing-rate activity (Matthews 2001). The simulation of excitatory cells also implemented a white noise process to reflect the spontaneous firing of pyramidal neurons (Deco et al. 2009). Inhibitory cells, however, captured the average activity of local pools of interneurons and hence were graded response neurons, not spiking cells. They also did not contribute to the white noise, as this stems from excitatory neurons. The synaptic plasticity was based on a classic Hebbian learning rule (Artola et al. 1990; Artola and Singer 1993) that has been supported extensively by empirical literature (Musso et al. 1999; Malenka and Bear 2004; Finnie and Nader 2012). This rule, which includes both LTP and homo- and hetero-synaptic LTD, is captured by the following definition of the change in synaptic weight Δ*w(i,j)* between a presynaptic cell *i* and a postsynaptic cell *j*:

$$\begin{align*} &\Delta w\left(i,j\right)\\&=\left\{\!\begin{array}{ll}+\Delta \kern0.33em if\kern0.33em {\omega}_E\left(i,t\right)\ge{\theta}_{pre}\kern0.33em and\kern0.33em V\left(j,t\right)\ge{\theta}_{+}& \!\!\!\!\!(LTP)\\{}-\Delta \kern0.33em if\kern0.33em {\omega}_E\left(i,t\right)\ge{\theta}_{pre}\kern0.33em and\kern0.33em {\theta}_{-}\le V\left(j,t\right)<{\theta}_{+}& \!\!\!\!\!\left( homosynaptic\kern0.33em LTD\right)\\{}-\Delta \kern0.33em if\kern0.33em {\omega}_E\left(i,t\right)<{\theta}_{pre}\kern0.33em and\kern0.33em V\left(j,t\right)\ge{\theta}_{+}&\!\!\!\!\!\left(heterosynaptic\kern0.33em LTD\right)\\{} 0\kern0.33em otherwise& \end{array}\right.\!\!\!\!\!\! \end{align*}$$

where $\theta$_pre_ ∈[0,1] is the minimum presynaptic activity required for LTP or homosynaptic LTD, $\theta$_−_ and $\theta$_+_ reflect postsynaptic membrane potential thresholds, *V(j,t)* is the membrane potential of cell *j*, and *ω_E_(i,t)* captures the estimated firing rate of cell *i* at time *t.* +Δ and − Δ (with Δ <  < 1 and fixed) reflect the model’s two possible synaptic efficacy changes, discretized from the continuous range of true possible changes.

#### System features

The functional properties of the model at the system level included a sparse and topographic connectivity structure of excitatory connections within and between areas, as well as local regulatory inhibition mechanisms within each area. For each network, the connections were initialized with random seeds. The initial connections between cells were established randomly except that they were confined to a topographic neighborhood, and the probability of a synapse being created was governed by a Gaussian function clipped to 0 outside the neighborhood, such that the probability decreased with increasing distance between cells. The weights of the synapses were also randomly initialized. The patchy and topographic nature of such a cortical connectivity structure has been supported by previous research (Amir et al. 1993; Kaas 1997; Braitenberg and Schüz 1998; Douglas and Martin 2004). Apart from the inhibitory links within a local neighborhood of neurons within an area (Fig. 1C), a global inhibition mechanism reflecting regulatory functions of subcortical structures (thalamus, basal ganglia) was realized (Braitenberg 1978; Yuille and Geiger 2003; Palm et al. 2014). The global inhibition mechanism was area-specific and served to keep activity in the network areas within physiologically acceptable levels. Note that global regulation mechanisms may serve as a mechanism of attention, as explored by earlier work (Deco and Rolls 2005; Garagnani et al. 2008). The full model specification can be found in appendix A and described in detail in previous simulation studies (Tomasello et al. 2018, 2019).

#### Area and connectivity structure

Each of the 12 cortical areas modeled in the neural network consisted of one layer of 625 excitatory cells, simulating spiking pyramidal neurons, and one layer of 625 inhibitory cells, simulating local pools of interneurons within the same cortical columns (Wilson and Cowan 1972; Eggert and van Hemmen 2000). The auditory subsystem of the modeled perisylvian regions included the primary auditory cortex (A1), auditory belt (AB), and parabelt (PB), and the articulatory subsystem included the inferior primary motor cortex (M1_i_), inferior premotor cortex (PM_i_), and inferior prefrontal cortex (PF_i_). The visual system was comprised of the primary visual (V1), temporo-occipital (TO), and anterior-temporal (AT) regions and the action system was composed of the dorsolateral primary motor (M1_L_), premotor (PM_L_), and prefrontal (PF_L_) areas. Each of these subsystems included an area considered to be a “connector hub,” also called a “convergence zone” (PB, PF_i,_ AT, and PF_L_) due to being a region with a high degree of connectivity and therefore multimodal integration (Damasio 1989; Pulvermüller 2013; van den Heuvel and Sporns 2013; Tomasello et al. 2017, 2018, 2019). The network’s connectivity structure between these areas is based on neuroanatomical evidence using diffusion tensor and diffusion-weighted imaging (DTI/DWI), summarized in Table 1. Generally, between-area connections included not only links between neighboring areas (black arrows in Fig. 1A and B) but also second-next-neighbor areas also called “jumping links” (blue arrows) and long distance cortico-cortical links (purple arrows), which have been shown to be vital to human language processing in previous simulation work (Schomers et al. 2017).

### Simulated learning

To simulate semantic learning, 22 networks, each thought to represent a different human cortex, were first created and randomly initialized and, subsequently, each presented with 18 different randomly generated input patterns. These input patterns represented 6 sensorimotor referent-related patterns, thought to convey perceptual information about 3 different visual objects and motor information about 3 manual actions, as well as 6 phonological word forms, each of which included an auditory and an articulatory activation pattern. Note that the total number of words used to teach each neural network was kept low, so as to match previous behavioral and neurocognitive studies investigating fast mapping that typically test infants with a low number of words (Carey and Bartlett 1978; Dollaghan 1985; Mills et al. 2005; Friedrich and Friederici 2011). Each input pattern was made up of 22 randomly selected cells from the 625 excitatory cells of a primary area (~3.5% of the cells). Although the inputs that real brains receive are not typically random and might have various structural patterns, we aimed to test the potential for semantic learning of *arbitrary* symbol-referent mappings without any added assumptions about specific input patterns. Future work could then build on this and investigate inputs with different structural properties and the effect this has on learning. The values in Table 2 describe the parameters used during all learning phases unless otherwise noted and were chosen on the basis of previous simulation studies (Garagnani et al. 2007, 2009; Garagnani and Pulvermüller 2011; Schomers et al. 2017; Tomasello et al. 2017, 2018, 2019).

**Table 2:** Parameter values. Model parameter values used in all networks during simulations.

***Simulation Parameter Values***	
Time constant (excitatory cells)	$\boldsymbol\tau$ = 2.5 (simulation time-steps)
Time constant (inhibitory cells)	$\boldsymbol\tau$ = 5 (simulation time-steps)
Total input rescaling factor	*k*_1_ = 0.01
Noise amplitude	*k*_2_ = 2^*^√(24/Δt)
Global inhibition strength	*k*_G_ = 0.70
Spiking threshold	*Thresh* = 0.18
Adaptation strength	$\alpha$ = 7.0
Adaptation time constant	$\boldsymbol\tau$ *_ADAPT_* = 10 (time steps)
Rate-estimate time constant	$\boldsymbol\tau$ *_Favg_* = 30 (time steps)
Global inhibition time constant	$\boldsymbol\tau$ *_GLOB_* = 12 (time steps)
*Postsynaptic Membrane Potential Values*	
	$\theta$ _+_ = 0.15
	$\theta$ _—_ = 0.14
*Presynaptic Output Required for LTP*	
	$\theta$ *_pre_* = 0.15
Learning Rate	Δ = 0.0012

#### Learning phase 1: forming phonological and referent representations

In this first learning stage, the referents and word forms were trained separately, mimicking object perception, action execution, and phonological learning without meaning as it may take place in the so-called babbling phase and during subsequent verbal repetition. To simulate perception of a referent object or the execution of a manual action, the network received visual object patterns to V1 or a manual action patterns stimulating M1_L_. All three non-relevant primary areas (M1_i_, A1, and M1_L_ for visual referents and M1_i_, A1, and V1 for action referents) received variable noise inputs that changed at each learning step to reflect uncorrelated inputs to these areas, which are typically present during learning and appear to be critical for preventing excessive cell assembly (CA) growth into adjacent regions (Doursat and Bienenstock 2006; Tomasello et al. 2019). For training the word forms, pairs of auditory and articulatory inputs were always presented simultaneously, with auditory input to A1 and articulatory input to M1_i_. This simulates the simultaneous articulatory and acoustic features activated when uttering a word (Baddeley 2003; Pulvermüller et al. 2014). When presenting word forms, both other primary areas (V1 and M1_L_) received the variable noise inputs.

On each learning trial, the referent or word form pattern was presented to the corresponding primary area(s) for 16 simulation time steps. This was followed by a period of no input (except for white noise), to allow the global inhibition to return to a baseline level prior to starting a new trial. This period lasted until the global inhibition level in selected regions (V1, M1_L_, A1, M1_i_, PF_L_, PB) fell below a predefined threshold of 0.75. During this learning stage, each referent and word form was presented 3,000 times. This value was chosen based on previous simulation studies (Garagnani and Pulvermüller 2016; Tomasello et al. 2017, 2018).

#### Learning phase 2: fast-mapping

After the model underwent the learning of referents and word forms separately, the same networks entered a secondlearning stage in which the learned object and action sensorimotor patterns were each paired to a learned word form pattern. This created 6 full semantic pattern sets in each network, each including a phonological word form and its meaning, grounded in sensorimotor referential information. To simulate the mapping between referents (object and action) and their learned auditory word labels, these were presented simultaneously. Therefore, for object words, input was given simultaneously to V1 and A1 and for action words to M1_L_ and A1. This captures the process of acquiring word meaning by hearing an object-related word while seeing a referent object or engaging in an action covered by a perceived action word (Tomasello and Kruger 1992; Vouloumanos and Werker 2009). Although many words are learned in the absence of their referents, this kind of direct embodied semantic learning is prominent in early language learning (Tomasello and Farrar 1986; Dunham et al. 1993; Houston-Price et al. 2006) and has also been documented to be relevant for rapid semantic learning, as shown, for example, by experimental studies in which participants were presented words auditorily while simultaneously being shown their referents (Carey and Bartlett 1978; Spiegel and Halberda 2011; Weismer et al. 2013; Merhav et al. 2015). Critically, this learning in early childhood appears to be purely associative and unsupervised, thus lacking explicit feedback. The absence of a need for explicit feedback calls for a simulation strategy avoiding feedback-based learning mechanisms and argues in favor of using a Hebbian learning rule. As in the first learning phase, variable uncorrelated inputs were presented to the irrelevant modality-preferential area (M1_L_ for object words, V1 for action words). No variable inputs were given in M1_i_, in order to avoid a delinking of the previously learned word form due to uncorrelated input and to allow for a reactivation of the full phonological representation from auditory input.

The structure of learning trials was the same as in the first learning phase: inputs were presented for 16 simulation time steps and trials started once global inhibition fell below the fixed threshold (for more details, see section above). Each full semantic pattern set, consisting of a referent and an auditory word input, was presented 100 times, and the progression of CA formation or the successful mapping between word form and referent was recorded time step by time step.

#### Simulating learning under high attention

To investigate the role of attention in fast word acquisition, we once again simulated the fast-mapping learning phase but with higher simulated attention during the first three learning episodes. This mimics high attention during the initial encounters with a novel word and referents, potentially promoting rapid word acquisition in these early presentations, after which attention returns to baseline. To realize high attention in the network, we lowered the global (area-specific) inhibition parameter (cortical activity regulation mechanism). Note again that inhibitory mechanisms have previously been discussed as a basis of attention to visual input and language (Deco and Rolls 2005; Garagnani et al. 2008). The global inhibition term was lowered from 0.70 to 0.50 for the first three presentations of the referent and auditory word input, so that weaker inhibition operated at the cortical neural level, thus reflecting greater computational resources and higher attention during the first word-referent encounters. After that, the inhibition parameter returned to the baseline value of 0.70 (Table 2, see appendix A for a formulation of how global inhibition was implemented). For direct comparison between high and low attention conditions, we took the same 20 networks that had already undergone the first learning stage and trained them on the fast-mapping phase in this high attention condition, again for 100 presentations each.

#### Simulating one-stage learning

To further test the importance of the pre-existing repertoire of referent and phonological representations for fast-mapping, we compared the two-stage learning process to a one-stage learning process in the same networks. To do so, we took all the models from the two-stage learning process for which we had the original states before any training and trained those identical models in a one-stage learning process, in which word-meaning mapping was simulated without any prior encounter with phonological and conceptual information. This way, the models had the same randomly initialized connectivity as well as the same randomly generated input patterns between the two learning processes, and all model parameters were kept the same. In one-stage learning, referents and word forms were not trained separately in an initial learning stage as in the fast mapping simulation. Instead, networks were presented with the word form and their paired sensorimotor referent input pattern simultaneously, right from the beginning of training, following the approach of previous work on associative semantic learning using these networks (Tomasello et al. 2017, 2018, 2019). For object words, input was given simultaneously to V1, A1, and M1_i_, and for action words, input was given to M1_L_, A1, and M1_i_. The non-relevant referent area received uncorrelated variable noise inputs. The structure of learning trials otherwise remained the same as in the two-stage learning process, and the formation of CAs was recorded at each time step using the same CA extraction process as for two-stage learning (described below). We did this on 13 networks, leaving 78 words or CAs to be analyzed per learning process. Note that due to an oversight in data storage, the one-stage learning was done with a smaller number of networks than in the fast mapping simulations (where 20 networks were used). However, given the low variance between the neural networks and the significant differences in learning between the two learning scenarios, a meaningful comparison can still be made between the two.

#### CA extraction

After each learning stage, we identified the distributed CAs that spontaneously emerged during learning. After the first learning stage, this involved quantifying the formed representations of visual objects, manual actions, as well as phonological word forms. These CA circuits were reactivated by simulating visual experience of an object by input to V1, performance of a manual action by input to M1_L_, or production of a word form, by simultaneous articulatory input to M1_i_ and auditory input to A1. No learning occurred during these simulations as they were only used to quantify the previously formed representations. To capture semantic circuits formed through the fast-mapping learning stage, CAs were quantified from just auditory input, simulating auditory word recognition after learning. This allowed us to examine whether the word form CAs had linked with the referent CAs sufficiently to lead to a distributed semantic representation that reactivated primary referent areas. Semantic associations were considered “linked” when at least 10% of the object recognition or action execution-related CA cells in the associated referent areas were reactivated from just auditory input to A1. This simulated word comprehension was done after each fast-mapping learning step, so that we could examine precisely when and to what extent mapping occurred. A “learning step” refers to all words being presented once, such that the number of “learning steps” mirrors the number of encounters with each word.

Each simulation for CA extraction involved the presentation of the corresponding input for 16 simulation time steps. No white noise was used during any of these simulations, as the networks were not in a learning phase and we wanted to avoid capturing noise in our quantification of the formed CAs. To capture the cells formally belonging to a CA, we used the same procedure applied in previous simulation work (Garagnani et al. 2008; Garagnani and Pulvermüller 2016; Schomers et al. 2017; Tomasello et al. 2017, 2018, 2019; Henningsen-Schomers and Pulvermüller 2021). During the simulation, the time-averaged firing rate of each excitatory cell was computed, and a cell was considered to belong to a CA circuit if this firing rate surpassed a particular threshold, specific to the area and CA. This threshold was defined as a fraction γ of the maximum time-averaged firing activity of any cell in that area in response to the pattern. Based on previous simulation work, we used γ = 0.5 (Garagnani et al. 2008, 2009; Tomasello et al. 2017, 2018). After quantifying the cells belonging to each CA, we computed the average number of cells in each area for each word type across all CAs in all networks. We gathered these averages to examine how the distributed semantic representations formed and developed during simulated fast-mapping.

#### Statistical analyses

After the first learning stage, we used a Poisson generalized linear model (GLM) with fixed effects of Area (levels: V1, TO, AT, PF_L_, PM_L,_ M1_L_, A1, AB, PB, PF_i_, PM_i_, M1_i_) and Referent Type (levels: Object vs Action), in order to examine differences in topography between visual object and manual action representations. After the second stage of learning (i.e. fast-mapping), to analyze the effects of word type and learning step on the topographical CA distributions, we performed a model comparison of a Poisson GLM and negative binomial GLM to select the most appropriate for our counts data. We analyzed all main and interaction effects of Word Type (levels: Object vs Action), Learning Step (from 0 to 100), and Area (levels: V1, TO, AT, PF_L_, PM_L,_ M1_L_, AB, PB, PF_i_, PM_i_, M1_i_) on the dependent variable, number of CA cells. Additionally, we performed a nonparametric aligned ranks transformation ANOVA using ARTool (Wobbrock et al. 2011) in order to examine the robustness of our results in an assumption-free context. All statistical analyses were done in R (R Core Team 2020) and negative binomial regression models were run using the MASS package (Venables and Ripley 2002). Unless otherwise stated, tests were two-tailed and an alpha value of 0.05 was used. Two networks were removed prior to statistical analysis due to merging of representations such that multiple CAs would coactivate and lead to excessive activation spreading, which rendered them impossible to analyze. This can be considered to reflect two subjects that failed to learn discriminable neural responses to the different semantic patterns. This resulted in 20 remaining networks, with 6 full semantic pattern sets each, yielding 120 CAs for analysis across fast-mapping steps, including 60 of each word type.

**Fig. 2:**
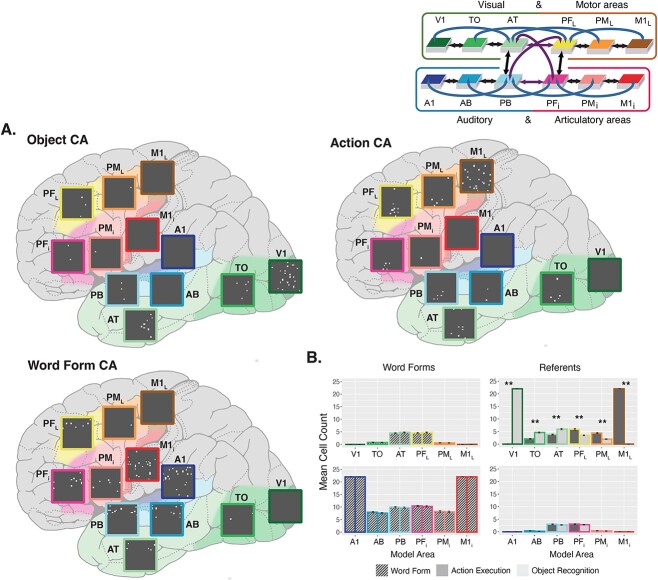
CAs formed prior to fast-mapping. **A**) CA examples. Each CA shows the cells belonging to it across the 12 areas, placed on a schematic illustration of the brain with the modeled cortical areas highlighted and color-coded. The color mapping between network areas and brain regions is shown in the schematic in the top right corner. Active cells are depicted as white dots in each gray area. Top-left is an example of the representation of a visual object referent. Top-right is an example of the correlates of a manual action execution. Bottom-left is an example of a phonological word form representation. **B**) Mean cell counts per area. CA cell counts shown in each area for word forms and referents averaged across 20 networks. Error bars depict standard error (SE). The colored outline of the bars maps them to their respective brain region. Cell counts for the object representations are shown in light gray and cell counts for action representations are shown in darker gray. Word form are later linked with either object or action representations and are split into separate bars on the basis of this future pairing, but the distinction is not meaningful at this point; hence, they all are depicted in gray with tilted stripes. In line with this, pairwise comparisons are only performed and shown for the referents. Depicted *P*-values have been Bonferroni-corrected. Asterisks illustrate significant differences between the number of neurons in CA circuits of object and action referents.

## Results

### Fast mapping—two stage learning

#### Learning phase 1

After the first learning stage, in which referents (object recognition and action execution) and phonological word forms were trained separately, we found stable, distinct topographical CA circuits that had spontaneously emerged during learning. Figure 2A shows an example CA distribution for each of the referent types, as well as a word form CA, depicted on a schematic illustration of the brain across the modeled cortical regions of the neural network. The average topographies of these circuits are shown in Fig. 2B. To examine the modality-specific topographies of visual objects and manual actions, we ran a Poisson GLM on CA cell counts with main and interaction effects of Referent Type (levels: Object, Action) and Area (levels: V1, TO, AT, PF_L_, PM_L,_ M1_L_, A1, AB, PB, PF_i_, PM_i_, M1_i_), which revealed a significant interaction between the two factors (χ^2^ (11) = 3825.60, *P* < 0.001). Pairwise comparisons between referent types in each area revealed significantly more cells belonging to object circuits in visual areas V1, TO, and AT, and significantly more cells belonging to action circuits in motor areas PF_L_, PM_L,_ and M1_L_ (all *P* < 0.001, Bonferroni-adjusted; Fig. 2B). Additionally, these referent-associated CAs reached all four connector hub regions (AT, PF_L_, PB, PF_i_) in all cases. Word form representations had the highest density of cells in the perisylvian language system but also reached all four connector hub regions central in the network architecture (Fig. 2B).

**Fig. 3:**
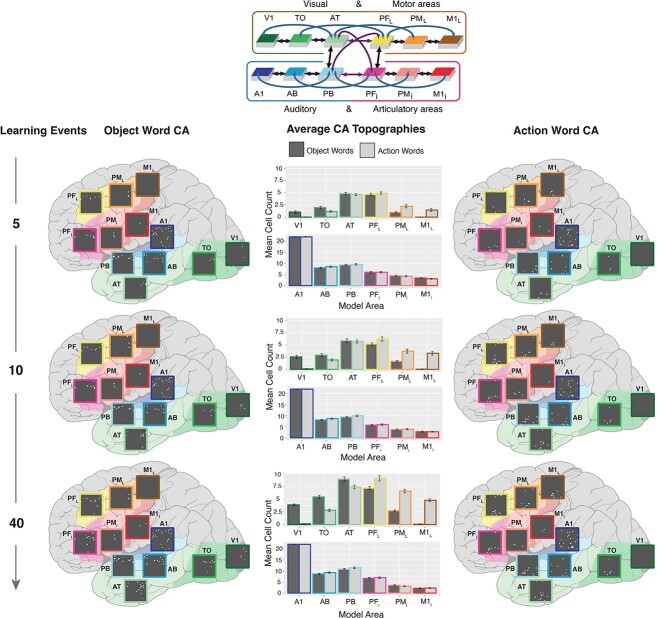
Interlinked cell assemblies for word forms and concepts (objects and actions) during fast-mapping. In the far-left and far-right columns, examples of CAs formation after 5 (top), 10 (middle), and 40 presentations (bottom) are shown. Each panel shows the cells activated by stimulating the auditory cortex, area A1 (dark blue), with one auditory word form pattern, which led to the activation of CAs spread across all 12 model areas. These activations are placed on a schematic illustration of the brain with the modeled cortical areas highlighted and color-coded. The color mapping between network areas and brain regions is shown in the schematic in the top-middle. Each gray box shows one simulated area of 625 excitatory cells, with active cells depicted as white dots. The three panels on the left show the CA development for an object word and those on the right that for an action word. Note the activity spreading into extrasylvian visual regions (AT, TO, and V1) for the object word and extrasylvian motor regions (PF_L_, PM_L_, and M1_L_) for the action word, showing successful category-specific fast-mapping. In the middle column, the bar plots show the CA cell counts in each area for action (dark gray) and object (light gray) words, averaged across 20 networks and 3 CAs of each word type per network. This is shown after 5 (top), 10 (middle), and 40 (bottom) learning trials. Error bars depict SE. The colored outline of the bars maps them to their respective brain region.

#### Learning phase 2—fast mapping

During the fast-mapping phase, CAs that had emerged across the different regions were determined after each learning step. Figure 3 shows an example of an object-word and action-word CA distribution, depicted in a schematic illustration of the brain, in response to auditory stimulation after 5, 10, and 40 learning trials of fast-mapping. This demonstrates the stronger reactivation of visual extrasylvian areas (V1, TO, and AT) in response to object words and motor extrasylvian areas (M1_L_, PM_L_, and PF_L_) in response to action words, reflecting linked referents and word forms. The bar plots depict the mean number of CA cells across the different cortical regions at different learning trials.

**Fig. 4:**
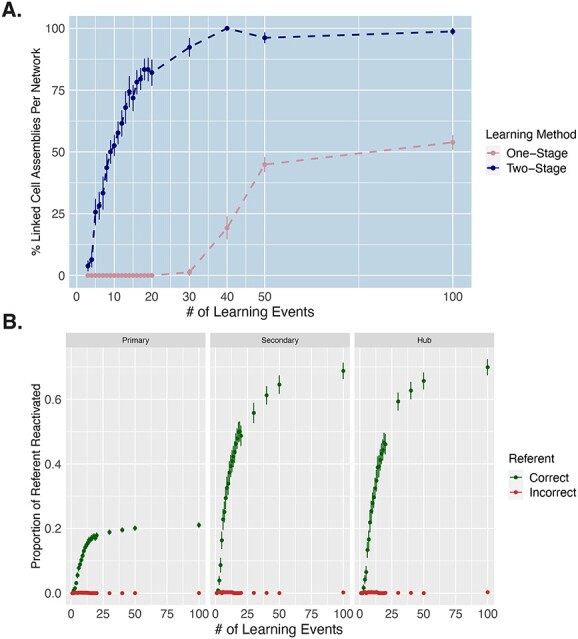
Rate of successful word-referent mapping. **A**) Percentage of rapidly mapped cell assemblies (CAs) across 100 learning trials in one-stage vs two-stage learning. The *y*-axis depicts, for each learning event, the percentage of CAs per network in which the word form was mapped sufficiently to the referent, allowing for reactivation of at least 10% of the object/action representation in referent extrasylvian areas (V1, TO, and AT for object words and M1_L_, PM_L_, and PF_L_ for action words) after auditory stimulation. This is computed across the different learning steps, on the *x*-axis. Error bars capture SE across networks. **B**) Proportion of correct vs incorrect referents reactivated across learning trials for two-stage learning. Green points depict the proportion of the *correct* referent representation that is reactivated after each learning event from just auditory stimulation. Red points depict the proportion of *incorrectly* activated referent representations, demonstrating that the networks did not make mapping errors. These results are separated by category-specific extrasylvian area, with “primary” corresponding to V1 for object words and M1_L_ for action words, “secondary” corresponding TO for object words and PM_L_ for action words, and “hub” corresponding to AT for object words and PF_L_ for action words. Error bars capture SE across CAs.

In order to investigate how rapidly word-referent mapping emerged, we examined the percentage of CAs that had formed an association between the word form and referent, after each presentation step. Successful linking of the word and referent representations was defined as the activation, upon stimulation with a word form pattern in A1, of at least 10% of the neurons constituting the correct referent representation in the associated referent areas (V1, TO, and AT for object words, and M1_L_, PM_L_, and PF_L_ for action words). Although white noise was always present during learning, we stopped this white noise during the CA extraction in order to ensure that any referent area activity in these CAs was not due to noise. Results of this analysis are plotted in Fig. 4A and show the percentage of CAs which mapped labels to their referents. This number increased monotonically across learning steps, with some mappings (5.83%) being achieved already after the first 3 learning events, the majority (55.8%) mapped by 10 learning events, and 70.0% mapped by 13 learning steps (Fig. 4A). We also show how the proportion of the referent representation being reactivated progressed across learning steps in each of the relevant referent areas (Fig. 4B). Importantly, we find that the proportion of incorrect referents being activated remains at 0 (Fig. 4B), showing that the networks rapidly learned the specific mapping of the words to their *correct* referent, and not just any object or any action representations.

#### Emergence of category-specificity during fast-mapping

To investigate the topographical formation of CAs for object and action words during fast-mapping across the different regions, we ran a Poisson regression on CA cell counts with main and all interaction effects of Word Type (levels: Object, Action), Area (levels: V1, TO, AT, PF_L_, PM_L,_ M1_L_, AB, PB, PF_i_, PM_i_, M1_i_), and Learning Step (0 to 100 steps). Counts in area A1 were removed from analysis due to zero variance across all conditions. This was expected due to stimulus input being presented to A1, resulting in full 22-cell activation in all cases. Due to evidence of overdispersion, quantified using a quasi-Poisson analysis that yielded a dispersion parameter of 1.48, we built a negative binomial GLM with the same formula. This was a significantly better fit to our data according to a likelihood ratio test, χ^2^ (1) = 259.74, *P* < 0.001, and therefore, the following results are from the negative binomial model. We used an additional nonparametric analysis with no assumptions regarding the distribution, which will be discussed after.

**Fig. 5:**
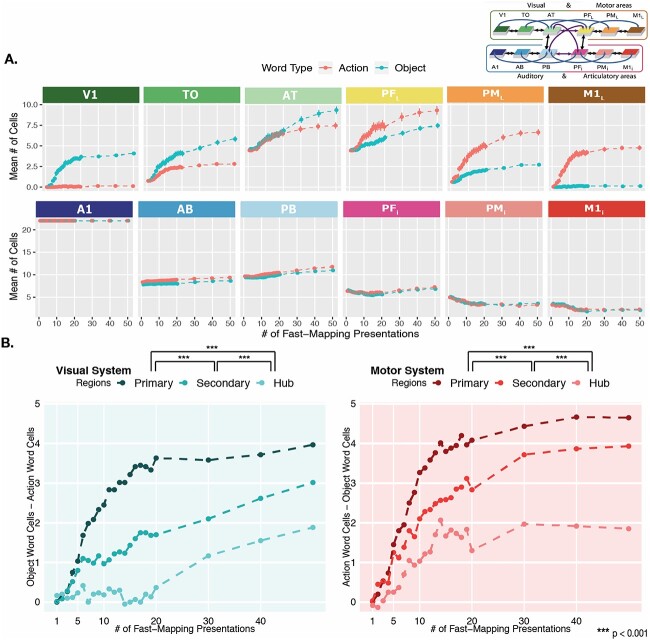
Category-specific topographies across fast-mapping. **A**) Mean action vs object word CA cells across areas. Emerging category-specific topographical distributions of CAs across learning steps. The *y*-axis captures the mean number of cells belonging to CAs of that word type, which are calculated specific to each area, at each learning step indicated on the *x*-axis. Learning steps run from 1 to 50 (with each vertical white line indicating 10). Error bars reflect SE. The schematic in the top-right corner showing the color-coded areas is adapted from Tomasello et al. 2018. **B**) Category-specificity between region types. The *y*-axes reflect the difference in mean number of CA cells between the two word types, which serves as a measure of the degree of category-specificity. The left plot in blue depicts the difference between object and action words in extrasylvian visual regions, with the primary region referring to V1, the secondary to TO, and the hub to AT. The right plot in pink depicts the difference between action and object words in extrasylvian motor regions, with the primary region referring to M1_L_, the secondary to PM_L_, and the hub to PF_L_. The significance indicators (^*^) at the legends refer to pairwise comparisons between area types collapsed across learning steps, and *P*-values have been Bonferroni corrected.

We expected that if our cortical network model and two-stage learning process could capture the rapid category-specific semantic learning associated with fast-mapping, we would find the number of CA cells associated with a word to depend on the word type and area, and for this to change across learning steps. Precisely in line with these predictions, our model yielded a significant 3-way interaction effect between Word type, Area, and Learning Step, (χ^2^ (10) = 65, *P* < 0.001), suggesting a change in the category-specific topographies over learning steps (Fig. 5A). Significant 2-way interactions were revealed between Word Type and Area (χ^2^ (10) = 10,963, *P* < 0.001) and between Area and Learning Step (χ^2^ (10) = 1517, *P* < 0.001), but not between Word Type and Learning Step (*P* = 0.77).

To investigate the obtained interaction between Word Type and Area and to examine emergent category-specific topographies across the network areas, we performed pairwise comparisons between word types within each area (excluding A1, for reasons stated above), collapsed across all learning steps. This revealed significant differences between word types in all extrasylvian areas, with more visual system activity associated with object words, and more motor system activity associated with action words (all *P* < 0.05, Bonferroni-adjusted; Fig. 5A). Significant differences between word types were unexpectedly found in two perisylvian areas: AB and PB. However, these differences were small (mDiff_AB_ = 0.69 cells, SE = 0.12; mDiff_PB_ = 0.44 cells, SE = 0.13) compared with the category-specificity of the extrasylvian regions across learning (Fig. 5A). The timing of category-specificity in extrasylvian regions was also investigated with a negative binomial GLM including factors of System (levels: Visual, Motor), Word Type, and Learning Step. Pairwise comparisons revealed category-specificity (captured by significant differences in activation between object and action words) to first emerge at 5 learning steps in the motor system and 6 learning steps in the visual system. These comparisons were Bonferroni-corrected. These early word category differences in CA topographies provide further evidence for successful and rapid mapping of phonological information onto semantic information after a small number of learning events.

The main results from this negative binomial GLM were also confirmed with a nonparametric analysis without any distributional assumptions. We performed a nonparametric aligned ranks transformation ANOVA with factors of Word Type, Area, and Learning Step and all main and interaction effects. This confirmed the robustness of our key finding of the 3-way interaction of the factors Word Type, Area, and Learning Step (*F*(230) = 7.10, *P* < 0.001).

In order to further investigate the emergence of category-specificity during fast-mapping, captured in the significant 3-way interaction effect, we ran a statistical analysis of the strength of category-specificity in the primary, secondary, and hub regions of the extrasylvian system across learning steps. We created a measure of the degree of category-specificity in each area type (primary, secondary, hub) by taking the mean difference in cell counts between object and action words per network in each extrasylvian area. For visual areas (V1, TO, AT), action word cells were subtracted from object word cells to capture the degree of specificity for object words, and for motor areas (M1_L_, PM_L_, PF_L_), object word cells were subtracted from action word cells to capture the degree of specificity for action words. We then built a linear regression model on the mean difference, including Area Type (levels: Primary, Secondary, Hub) and Learning Step as main effects and their interaction effect. This revealed a significant interaction between Area Type and Learning Step (*F*(2) = 22.68, *P* < 0.001)*,* suggesting that the area type modulated the degree of category-specificity that emerged over learning steps, with category-specificity emerging the fastest in the primary, followed by the secondary and finally in the hub regions. Pairwise comparisons collapsed across 100 learning steps revealed a significantly stronger degree of category-specificity in the primary regions than either secondary regions or hubs, as well as a significantly stronger degree of specificity in secondary regions compared with hubs (all *P* < 0.001, Bonferroni-adjusted; Fig. 5B).

#### Fast mapping under high attention

We also investigated if a high attention level during initial word-referent exposures (learning phase 2) would increase the learning speed of semantic associations. To analyze the putative influence of attention on model performance, we added the attention condition described in *Methods* along with a further factor (±Attention) to our previous negative binomial model on cell counts, such that the full model now included all main and interaction effects of Attention (2 levels), Area (11), Word Type (2), and Learning Step (100). The attention conditions consisted of High Attention, in which the global inhibition parameter was lowered to 0.50 for the first three presentations during the fast-mapping learning stage and then returned to 0.70, and Baseline Attention, in which global inhibition was kept at the baseline level of 0.70 throughout fast-mapping (for more detail see Methods section).

**Fig. 6:**
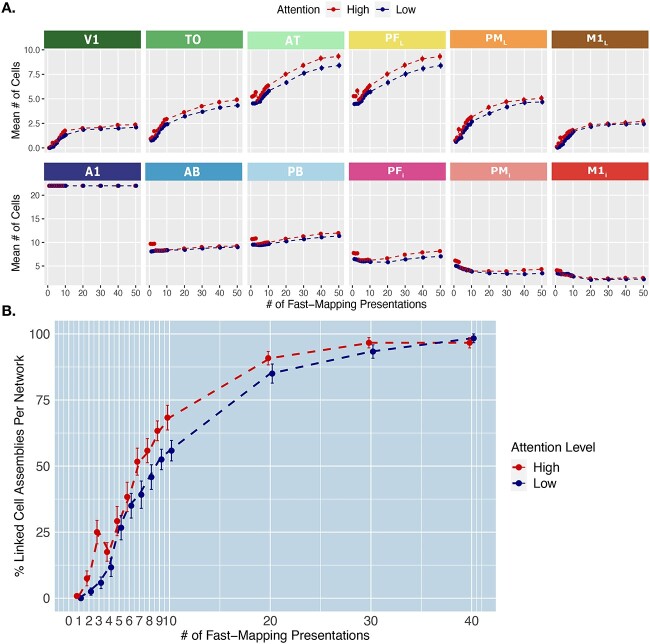
Effect of attention on fast-mapping. **A**) Mean cell counts per area and attention condition across learning. Emerging distributions of cell counts across learning steps in each attention condition. The *y*-axis captures the mean number of cells belonging to CAs in each area, collapsed across word-types, at each learning step on the *x*-axis. The baseline-attention condition reflects the baseline condition in which global inhibition is kept at 0.70 throughout learning (blue line). The high-attention condition reflects a condition in which a global inhibition of 0.50 for the first three learning steps, and then raised to baseline (0.70, red line). Error bars reflect SE. **B**) Rate of linking per attention condition. The *y*-axis depicts the percentage out of the CAs per network that have linked such that auditory input reactivates at least 10% of the referent representation in the associated extrasylvian areas (V1, TO, and AT for objects and M1_L_, PM_L_, and PF_L_ for action words). This is computed across the different fast-mapping learning steps, on the *x*-axis. Error bars reflect SE.

We found the same significant 3-way interaction between Area, Word Type, and Learning Step as in our previous analysis, but no significant 4-way interaction including Attention (*P* = 0.98). However, we found significant 3-way interactions between Attention, Area, and Learning Step (χ^2^ (10) = 25, *P* = 0.0047), as well as between Attention, Word Type and Area (χ^2^ (10) = 49, *P* < 0.001). The latter indicates that the attention condition modulated the overall category-specific topographies of CAs, but not in a way that depended on the learning step. The interaction between Attention, Area, and Learning Step also suggests that attention had a general influence on the spread of activity in particular areas across fast-mapping (Fig. 6A). We also compared the percentage of CAs that had linked their word form and referent representations (defined above) between attention conditions across learning steps. The influence of high attention in increasing the linking rate can be seen in Fig. 6B. Taken together, these results suggest that increased attention during early stages of word exposure promotes the spread of activity and hence word learning via fast-mapping, and this influence does not interact with the learning step but has an overall effect across learning.

#### One-stage learning results and comparison with two-stage learning

To directly assess the importance of prior knowledge for the rate of word meaning acquisition in the two-stage learning, we simulated direct semantic associative learning, which we refer to as a one-step learning process. In other words, this captures a learning scenario in which the brain has no previously formed phonological and conceptual representations prior to associative semantic learning, during which the referent and word form are then experienced at the same time. Specifically, one-stage learning was simulated by simultaneously giving three input patterns in three primary regions of the model (A1 and M1_i_ for word forms and either V1 for object or M1_L_ for action referents) from the beginning of training to capture the same word learning processes as in previous work (Tomasello et al. 2017, 2018, 2019). All other elements of the training were the same as in the two-stage learning (see Methods section for more detail). Just like in two-stage learning, we stopped the networks after each learning step and extracted emerging CAs following the same procedure (auditory word recognition) as for the fast-mapping simulation. We then investigated how rapidly learning (i.e. CA formation) occurred under one-stage learning (direct word-meaning mapping) compared with the two-stage learning (fast-mapping). In both cases, a word was considered to be linked with its referent when at least 10% of the referent representation was reactivated in the associated extrasylvian areas (V1, TO, and AT for object words, and M1_L_, PM_L_, and PF_L_ for action words) from just auditory input. Because the very first inputs into networks that have not yet received any other training can lead to explosive bursts of activity, which are not indicative of learning and are not interpretable, we removed the first two learning trials from both learning processes for this analysis. These results are shown in Fig. 4A, revealing the learning rate in one-stage learning to be substantially slower than two-stage learning, with less than 25% of words successfully learned by 40 learning trials in one-stage learning, compared with 98% in two-stage learning. Also note that in one-stage learning, after 100 learning events, only 50% of the words have been successfully learned (i.e. reactivate the referent representation).

## Discussion

This simulation study aimed to investigate the biological principles underlying fast-mapping in word meaning acquisition using biologically constrained neural networks. We focused on capturing mechanisms of rapid word-meaning mapping from hearing a word while either seeing its referent object or performing its related action, as it has been documented in studies of early semantic learning (Tomasello and Farrar 1986; Tomasello and Kruger 1992; Dunham et al. 1993; Houston-Price et al. 2006; Vouloumanos and Werker 2009). Our model results show that after an initial step of learning a repertoire of referential and phonological representations, associative learning of word-meaning mappings by unsupervised Hebbian mechanisms is surprisingly fast. Most distributed semantic representations of object and action words formed within less than 10 co-presentations of a word and its referent, with the first successful form-meaning linkages even emerging in the first few learning events, and a mean of 26.67% (SE = 4.59) of the symbolic links per network being made after just 5 presentations (Fig. 4A). In striking contrast to this two-stage learning regime, in a one-stage learning process without prior exposure, form-meaning linkages did not start emerging until after 30 learning events and only reached a mean of 53.85% (SE = 2.77) of the links being successfully formed after 100 presentations (Fig. 4A). Interestingly, the fast-mapping mechanism does not seem to require attentional mechanisms but can be modulated by them, particularly if high levels of attention are implemented across simulated cortical areas. The emergent semantic circuits showed category-specific topographical distributions, with more activity in the visual system in response to object words and more activity in the motor system in response to action words, replicating previous simulation studies (Tomasello et al. 2017, 2018). Examining the role of the convergence hub areas, we observed that all referent and word form representations activated at least some cells in all hub regions after the first learning stage, highlighting the potential role of these hubs in promoting rapid linking of information from different modalities. Below we discuss the results in light of previous empirical evidence, fast-mapping theories, and novel predictions.

### The neurobiological principles of fast-mapping in word acquisition

Childrens’ vocabulary at an early stage of language development can already be vast, and, intriguingly, some of those symbols are rapidly acquired from few exposures. Therefore, many researchers consider fast-mapping a crucial learning mechanism for language acquisition (Heibeck and Markman 1987; von Koss Torkildsen et al. 2008). Although neural correlates of rapid word-meaning mapping have been reported (Friedrich and Friederici 2011; Hofstetter et al. 2017; Vukovic et al. 2021), putative neuromechanistic explanations at the neuronal and cortical level are still lacking (Davis and Gaskell 2009).

In the present study, we show that a spiking neural network equipped with Hebbian correlation learning and constrained by cortical neuroanatomy can offer a neuromechanistic explanatory account of fast mapping based mainly on two prerequisites:

(i) **Pre-existing framework of neural representations:** Knowledge about objects, actions, and categories into which these can be grouped is typically acquired prior to the semantic association between representations (Quinn et al. 1993; Mareschal and Quinn 2001; Mayor and Plunkett 2010; Westermann and Mareschal 2014; Atir-Sharon et al. 2015; Vasilyeva et al. 2019). Likewise, the articulations spontaneously produced by the infant during the so-called babbling stage include ample evidence for the building of language-specific phonological and syllabic representations before they are being linked to conceptual knowledge (Vihman et al. 1985; Werker and Tees 1999; Werker and Hensch 2015). In the present model, this scenario led to the formation of stable, strongly connected, distributed CAs, whose formation is driven by activity patterns in visual, motor, or auditory areas. These CAs represent objects, actions, and phonological tokens, respectively. Intriguingly, these specific neural representations all extended into the connector hub regions central to the neural architecture (see Fig. 1) which can be described as convergence zones (Damasio 1989) for integrating information across modalities and domains.
(ii) **Persistent neural activation:** Co-activation of the pre-acquired neural representations in simulated associative semantic learning led to rapid strengthening of the available synaptic links between form and meaning circuits after only a few learning events. Note also that the number of CA cells was generally largest in connector hub regions AT, PF_L_, PB, PF_i_ (aside from the stimulated primary areas) and, upon stimulation, a large number of cells were constantly active there, thus prolonging the time of co-activity and making these areas ideal sites for interlinking different CAs. These model results are in line with experimental work documenting the role of convergence regions in fast-mapping (Atir-Sharon et al. 2015).

The fast-mapping learning process simulated here is consistent with established two-stage theories of semantic learning with an initial stage of development in perceptual systems for parsing referent categories and speech, followed by a semantic learning stage in which labels are mapped rapidly to referents (MacNamara 1972; Markman 1992; Mayor and Plunkett 2010; Sharon et al. 2011; Smith et al. 2014; Westermann and Mareschal 2014; Atir-Sharon et al. 2015). Work on visual processing demonstrates that the ability to recognize visual objects and to form visual categories develops independently of language, already in 3-month-old infants (Quinn et al. 1993, 1997; Mareschal and Quinn 2001; Quinn and Schyns 2003; Westermann and Mareschal 2014). Likewise, there is evidence for acquisition of knowledge about linguistic phonological forms independently from concepts or meaning. At the babbling stage toward the end of the first year, language-specific phonological knowledge becomes manifest in the infant’s output (Vihman et al. 1985; Werker and Tees 1999; Werker and Hensch 2015) and, in close temporal vicinity, phonetic discrimination and categorization can be demonstrated in speech perception (Werker and Tees 1999; Tsao et al. 2004; Kuhl et al. 2005; Werker and Hensch 2015). Further studies have shown that infants can store whole word forms as early as 8 months of age (Jusczyk and Hohne 1997), and such word storage occurs in accordance with statistical properties of the lexicon (Estes et al. 2007). Furthermore, mathematical modeling of vocabulary exposure in infants suggests that by 18 months children may have even a few thousand whole words in their lexicon, without necessarily yet associating them with any meaning (Swingley 2007). Based on these lines of evidence, it is highly plausible that a child has already formed categories for visual items and has built full phonological representations of syllables and possibly word forms through babbling and subsequent mimicking of adults’ utterances (Pulvermüller and Schumann 1994; Westermann and Reck Miranda 2004) prior to learning links between objects and labels. These preliminary cognitive representations of concepts and phonological forms may greatly facilitate the semantic mapping of form and meaning. Here, we go a step further than cognitive theories just postulating form-meaning linkage, by discussing how such a two-stage fast-mapping semantic learning mechanism may operate mechanistically at the synaptic, cellular, neural circuit, and cortical area levels.

Critically, this work shows that brain-constrained neural networks equipped with biologically realistic Hebbian learning can explain the formation of symbolic representations after less than 10 learning events (Fig. 4A). This goes against criticisms that Hebbian learning may not be able to explain such rapid plasticity, as it involves incremental modification of synaptic weights so that numerous learning events are often used to produce reliably active cell assemblies (Garagnani and Pulvermüller 2016; Tomasello et al. 2017, 2018; Henningsen-Schomers and Pulvermüller 2021). Here, we show that after initial formation of conceptual and phonological circuits, which also maintain activation for some time, the mapping between form and meaning representations is more efficient as compared with models simulating word learning without prior learning of relevant knowledge (see Fig. 4A). Hence, the present results may contribute to a mechanistic explanation of how infants acquire language in the early stage of language development. Note that the acquisition of word meanings in infants could be a combination of a two-step and a one-step learning process. Here, we show that the speed of learning differs substantially between the two learning scenarios, thus suggesting that the availability of pre-acquired knowledge plays a critical role for the rate of semantic associative learning.

We also investigated whether attentional mechanisms are crucial and thus required for rapid word-meaning mapping (Regier 2005; Mayor and Plunkett 2010) or whether such mapping is an automatic and implicit process not requiring attention to be directed toward the stimuli (Shtyrov et al. 2010; Shtyrov 2011; Atir-Sharon et al. 2015; Merhav et al. 2015; Partanen et al. 2017; Vasilyeva et al. 2019). Our findings support the latter claim but also confirm the importance of attention. We reveal that high attention during initial encoding of the word-referent association speeds up the mapping process. However, we also demonstrate that fast-mapping occurs with baseline attention levels throughout learning and that the influence of attention may be driven by a generally enhanced activity level across all areas. It remains to be investigated whether a more focal attention modulation (dedicated, for example, to word form or semantic processing) will similarly modulate the speed of fast mapping.

The present findings also build on previous simulation work examining the emergence of category-specific semantic processing during associative learning (Garagnani and Pulvermüller 2016; Tomasello et al. 2017, 2018), which had yet to be explored in the context of fast-mapping. In line with these studies, we show that the co-occurrence of object- or action- with word-form-related information leads to the formation of semantic circuits with category-specific topographical distributions, reaching into motor and visual areas for action- and object-related words, respectively. Here, we demonstrated that such category-specificity can emerge rapidly during fast-mapping, and furthermore, we examined how it develops across learning. This revealed category-specific processing to emerge first in primary areas, followed by secondary and hub regions where it remained the least pronounced. Importantly, despite showing weaker category-specificity, the activity in all of the investigated connector hub regions increased substantially during fast-mapping. It therefore appears that they became prime sites of mixing and binding of semantic information (van den Heuvel and Sporns 2013). Taken together, this supports semantic theories postulating multiple semantic hubs for general form-meaning binding along with modality preferential regions for category-specific semantics (Binder and Desai 2011; Pulvermüller 2013).

The present results offer a biological mechanistic explanation for category-specific fast mapping at the cellular and cortical levels, by means of a brain-constrained neural network. We believe that this work may also have implications for other modeling pursuits within fields such as robotics or natural language processing. Progress in robotics and the spreading opportunity for human–robot interaction has highlighted the need for embodied robot language models that can tackle the symbol grounding problem (Harnad 1990; Cangelosi 2006; Raggioli and Cangelosi 2022). Our model may be relevant to this endeavor, by revealing a simple mechanism that can account for grounded semantic learning of both object and action words, and can do so in an implicit, unsupervised way that centers around the model’s own experiences and principles, as also suggested by previous word-meaning acquisition work with robots (Salvi et al. 2012). The potential applicability of these brain-constrained network models to robots was suggested in a previous study using a much simpler, nonspiking model with 6 areas that served as a controller after learning (Adams et al. 2014). This work also contributes directly to the push to derive architectures and inspiration for robot models from neurophysiology and cognitive neuroscience (Madden et al. 2010; Salvi et al. 2012). Such cooperation between fields has also been critical for progress in deep learning (Lake et al. 2017; Hassabis et al. 2017; Pulvermüller et al. 2021), and our model could be particularly relevant to multimodal deep learning models that integrate information from different modalities (Summaira et al. 2021). Principles uncovered here, such as the role of attentional and regulatory mechanisms (local and global inhibition), the importance of central hub regions with increased connectivity based on neuroanatomical evidence, the need for noise to allow meaningful learning, and the influence of modality-specific pretraining on learning efficiency, among others (see Pulvermüller et al. 2021 for a discussion), might be informative for future deep learning approaches.

Finally, we discuss limitations and perspectives of this work. Somewhat contrasting with the two-stage learning assumption (Markman 1992; Regier 2005; Mayor and Plunkett 2010; Westermann and Mareschal 2014), the phonological representations built during babbling or imitation may not always yield full word form representations, but just phoneme and syllable circuits. In some cases, word form circuits may need to be developed from more basic phonological representations, and this may happen in parallel with the semantic conceptual mapping. The impact on the speed of acquisition in this learning situation may show further critical insights on word-meaning acquisition, which could be the focus of further simulations, along with other training protocols such as phonological learning from just auditory inputs alone. Further model extensions could also address other forms of semantic knowledge acquisition, for instance, from text or variable context, in which semantic mapping might be explained similarly to this work, from coactivation of linguistic representations. Finally, the model takes critical steps with regard to biological realism and offers a novel mechanistic account for fast mapping and language processing, potentially setting the stage for reaching human brain-like performance in the rapid learning of much larger numbers of words. It could also be the subject of further studies to replicate the results with thousands of words in a much larger network, by expanding the number of areas, connections, as well as the amount of neural material in the network architecture. The present work is only an initial step toward exploring the material basis of rapid language and symbolic learning in brain-constrained neural networks, but an important one for better understanding the mechanisms underlying it. Besides the possible explanatory implication in using such models, this work can also pave the way for investigating neuroplasticity after specific cortical area lesioning or deprivation, such as simulating language acquisition and processing in blind people (see previous simulation on this by Tomasello et al. 2019) and possibly have a more practical implication by using the predictions of the model to inform neurosurgical planning (for discussion see Picht et al. 2021).

In summary, “fast-mapping,” the rapid semantic interlinking of word forms and their meaning, has been observed in early childhood and investigated in behavioral and neuroimaging studies. It is commonly agreed that it represents a vital mechanism for language acquisition. Here, we studied the neurobiological mechanisms underlying fast-mapping by simulating it using a neurobiologically constrained neural network model. Our results demonstrate that, within a model replicating major aspects of the structure and connectivity of relevant modality-preferential and modality-general areas, a two-stage learning process driven by realistic Hebbian learning is sufficient to account for rapid semantic interlinking of phonological and conceptual circuits. One-stage associative learning, in which the model was not exposed to any prior phonological or conceptual knowledge, showed learning success only much later, demonstrating gradual semantic learning. Furthermore, our results suggest that attention facilitates but is not strictly necessary for fast-mapping.

## Data and materials availability

Raw data is publicly available under https://osf.io/faj3p/.

## Appendix A

### Structure and function of the spiking model

Each of the 12 simulated areas is implemented as two layers of artificial neuron-like elements (“cells”), 625 excitatory and 625 inhibitory, thus resulting in 15,000 cells in total. Each excitatory cell “*e*” consists of a leaky integrate-and-fire neuron with adaptation and simulates a single pyramidal cell representative of excitatory spiking activity in a cortical microcolumn, while its twin inhibitory cell “*i*” (see Fig. 1C) is a graded-response cell simulating the average inhibitory response of the cluster of interneurons situated in a local neighborhood (Wilson and Cowan 1972; Eggert and van Hemmen 2000). The state of each cell *x* is uniquely defined by its membrane potential *V*(*x,t*), specified by the following equation:

(A1.1) \begin{equation*} \tau \cdotp \frac{dV \left(x,t\right)}{dt}=-V\left(x,t\right)+{k}_1\left({V}_{In}\left(x,t\right)+{k}_2\eta \left(x,t\right)\right) \end{equation*}

where *V_In_* (*x,t*) (defined by equation A1.2) is the net input acting upon cell *x* at time *t* (sum of all inhibitory and excitatory postsynaptic potentials—I/EPSPs; inhibitory synapses are given a negative sign), *τ* is the membrane’s time constant, *k*_1_, *k*_2_ are scaling values (see Table 2 for the specific parameter values used in the simulations) and *η*(*·*,*t*) is a white noise process with uniform distribution over [−0.5,0.5]. Note that noise is an inherent property of each model cell, intended to mimic the spontaneous activity (baseline firing) of real neurons. Therefore, noise was constantly present in all areas, in equal amounts (inhibitory cells have *k*_2_ *=* 0, i.e., the noise is generated by the excitatory cells in the model for convenience).

(A1.2) \begin{equation*} {V}_{In}\left(x,t\right)=-{k}_G{\omega}_{\text{G}}\left({A}_x,t\right)+\sum_{\forall \text{y}}{w}_{\text{x},\text{y}}\cdotp \phi \left(y,t\right) \end{equation*}

In Equation (A1.2), *y* varies over all cells in the network, *w*_*x*,*y*_ is the weight of the link from *y* to *x*, and $\phi$ (*y*,*t*) is *y*’s current output (1 or 0), as defined below (A2); ω*_G_*(*A_x_*,*t*) is the area-specific (or “global”) inhibition for area *A* where cell *x* is located (see explanation below and Eq. A3.3): this term is identical for all excitatory cells *x* in *A* and absent for inhibitory cells (*k_G_* is as scaling constant). The weights of inhibitory synapses are assigned a negative sign. The output (or transformation function) *ϕ* of an excitatory cell *e* is defined as follows:

(A.2) \begin{equation*} \phi \left(e,t\right)\left\{\begin{array}{ll}\ 1\kern0.50em \textrm{if}\ \left(V\left(e,t\right)-\alpha \omega \left(e,t\right)\right) > \textrm{thresh}\ \\{}\ 0\ \ {otherwise}\ \end{array}\right. \end{equation*}

Thus, an excitatory cell *e* spikes (=1) whenever its membrane potential *V*(*e,t*) overcomes a fixed threshold *thresh* by the quantity *αω.*(*e,t*) (where *α* is a constant and *ω* is defined below). Inhibitory cells are graded response neurons as they intend to represent the average impact of a cluster of local interneurons; the output *ϕ*(*i,t*) of an inhibitory neuron *i* is 0 if *V*(*i,t*) < 0 and *V*(*i,t*) otherwise.

To simulate neuronal adaptation (Kandel et al. 2012), function *ω*(*·,t*) is defined so as to track the cell’s most recent firing rate activity. More precisely, the amount of adaptation *ω*(*e,t*) of cell *e* at time *t* is defined by:

(A.3.1) \begin{equation*} {\tau}_{ADAPT}\cdotp \frac{d\omega \left(e,t\right)}{d t}=-\omega \left(e,t\right)+\phi \left(e,t\right) \end{equation*}

where is the “adaptation” time constant. The solution *ω*(*e,t*) of Eq. (A3.1) is the low-pass-filtered output *ϕ* of cell *e*, which provides an estimate of the cell’s most recent firing-rate history. A cell’s average firing activity is also used to specify the network’s Hebbian plasticity rule (see Eq. (A4) below); in this context, the (estimated) instantaneous mean firing rate *ω_E_*(*e*,*t*) of an excitatory neuron *e* is defined as:

(A.3.2) \begin{equation*} {\tau}_{Favg}\cdotp \frac{d{\omega}_E\left(e,t\right)}{dt}=-{\omega}_E\left(e,t\right)+\phi \left(e,t\right) \end{equation*}

Local (lateral) inhibitory connections (see Fig. 1C) and area-specific inhibition are also implemented, realising, respectively, local and global competition mechanisms (Duncan et al. 1997; Duncan 2006). More precisely, in Eq. (A1.2) the input *V_In_*(*x,t*) to each excitatory cell of the same area includes an area-specific (“global”) inhibition term *k_G._ω_G_*(*e*,*t*) (with *k_G_* a constant and *ω_G_*(*e*,*t*) defined below) subtracted from the total I/EPSPs postsynaptic potentials *V_In_* in input to the cell; this regulatory mechanism ensures that area (and network) activity is maintained within physiological levels (Braitenberg and Schüz 1998):

(A3.3) \begin{equation*} {\tau}_{GLOB}\cdotp \frac{d{\omega}_G\left(e,t\right)}{dt}=-{\omega}_G\left(e,t\right)+\sum_{e\in \textrm{area}\ }\kern0.1em \phi \left(e,t\right) \end{equation*}

Excitatory links within and between (possibly non-adjacent) model areas are established at random and limited to a local (topographic) neighborhood; weights are initialized at random, in the range [0, 0.1]. The probability of a synapse to be created between any two cells falls off with their distance (Braitenberg and Schüz 1998) according to a Gaussian function clipped to 0 outside the chosen neighborhood (a square of size *n* = 19 for excitatory and *n* = 5 for inhibitory cell projections). This produces a sparse, patchy and topographic connectivity, as typically found in the mammalian cortex (Amir et al. 1993; Kaas 1997; Braitenberg and Schüz 1998; Douglas and Martin 2004).

The Hebbian learning mechanism implemented simulates well-documented synaptic plasticity phenomena of long-term potentiation (LTP) and depression (LTD), as implemented by Artola, Bröcher and Singer (Artola et al. 1990; Artola and Singer 1993). In the model, we discretize the continuous range of possible synaptic efficacy changes into two possible levels, +Δ and − Δ (with Δ <  < 1 and fixed). Following Artola et al., we defined as “active” any (axonal) projection of excitatory cell *e* such that the estimated firing rate *ω_E_*(*e*,*t*) of cell *e* at time *t* (see Eq. A3.2) is above ϑ_pre_, where ϑ_pre_∈[0,1] is an arbitrary threshold representing the minimum level of presynaptic activity required for LTP to occur. Thus, given a pre-synaptic cell *i* making contact onto a post-synaptic cell *j*, the change Δ*w*(*i,j*) inefficacy of the (excitatory-to-excitatory) link from *i* to *j* is defined as follows:

(A4) \begin{align*} & \varDelta w\left(i,j\right)= \cr & \left\{\begin{array}{lll}+\varDelta & if{\omega}_E\left(i,t\right)\ge{\theta}_{pre}\kern0.33em and\kern0.33em V\left(j,t\right)\ge{\theta}_{+}\kern0.50em \ \kern0.50em \ \kern0.50em & (LTP)\\{}-\varDelta & if{\omega}_E\left(i,t\right)\ge{\theta}_{pre}\kern0.33em and\kern0.33em {\theta}_{-}\le V\left(j,t\right)<{\theta}_{+}& \left( homosynaptic\ LTD\right)\\{}-\varDelta & if{\omega}_E\left(i,t\right)<{\theta}_{pre}\kern0.33em and\kern0.33em V\left(j,t\right)\ge{\theta}_{+}& \left( heterosynaptic\ LTD\right)\\{}0& otherwise& \end{array}\right. \nonumber\\ \end{align*}
